# IgE-Mediated Allergy and Asymptomatic Sensitization to Cannabis Allergens—Review of Current Knowledge and Presentation of Six Cases

**DOI:** 10.3390/medicina60060954

**Published:** 2024-06-08

**Authors:** Jakub Wąsik, Aleksandra Likońska, Marcin Kurowski

**Affiliations:** 1 Department of Immunology and Allergy, Medical University of Lodz, 90-419 Lodz, Poland; jakub.wasik1@student.umed.lodz.pl (J.W.); aleksandra.likonska@umed.lodz.pl (A.L.); 2 Student Scientific Association, Department of Immunology and Allergy, Medical University of Lodz, 90-419 Lodz, Poland

**Keywords:** cannabis allergy and sensitization, Can s 3, component-resolved diagnostics, cross-reactivity, anaphylaxis, IgE

## Abstract

Cannabis allergy is a relatively new phenomenon described in the 1970s. Its increased frequency has been observed over the last years due to the increasing therapeutic and recreational use of cannabis-based products. Sensitization possibly leading to allergy symptoms can occur not only through the smoking of cannabis, but also through ingestion, the inhalation of pollen, or direct contact. The severity of symptoms varies from benign pruritus to anaphylaxis. There is scant information available to support clinicians throughout the entire therapeutic process, starting from diagnosis and ending in treatment. In this review, we present six cases of patients in whom molecular in vitro testing revealed sensitization to cannabis extract and/or cannabis-derived nsLTP molecules (Can s 3). Based on these cases, we raise important questions regarding this topic. The article discusses current proposals and highlights the importance of further research not only on cannabis allergy but also on asymptomatic sensitization to cannabis allergens, which may be ascertained in some percentage of the population.

## 1. Introduction

*Cannabis sativa* L. is an annual plant of the Cannabinaceae family used in many branches of industry, and also for recreational and medical purposes. Cannabis-based medicines are effective in patients with several conditions, including multiple sclerosis, chronic pain, and in palliative medicine, yet not without adverse events [1]. Their main natural components include phytocannabinoids (containing the key psychoactive compound, Δ-9-tetrahydrocannabinol (D9-THC)) and terpenoids [2]. In 2021, 219 million people used cannabis (4% of the global adult population), making it the world’s most commonly used drug [3].

Despite such wide use, there is little research on the cannabis potential of sensitization eliciting hypersensitivity reactions. The first case report of a marijuana allergy was reported more than half a century ago, in 1971 [4]. Until now, the majority of research on possible hypersensitivity to marijuana has focused on its immunopathogenesis. No guidelines regarding a diagnosis or treatment of marijuana hypersensitivity have been issued to date.

The fact that cannabis allergy (CA) is not uncommon in everyday clinical practice is confirmed by the results of a survey among allergists. Among 445 respondents, 192 (43.1%) have seen patients with suspected CA [5]. Moreover, 10 of the 401 (2.5%) noncurrent cannabis users reported an allergy to cannabis. These figures may vary from country to country, depending on the legal status of recreational cannabis use. Nevertheless, in recent years there has been an increase in products based on its ingredients, such as cannabidiol (CBD) oils.

The aim of our paper is to acquaint readers with the current literature on cannabis sensitization and allergy. In the section below, to introduce the topic of this review, six cases of sensitization to cannabis allergen will be presented. They reflect clearly that it occurs in patients of different ages and different backgrounds. The variety of symptoms, ranging from asymptomatic sensitization to severe systemic reactions, shows the complexity of the topic. Then, the entire diagnostic and therapeutic process will be discussed in detail, starting with the pathogenesis and ending with the search for possible therapeutic agents. The cases discussed below also present aspects that require further research, for example, possible cross-reactions between cannabis allergens and allergens belonging to other groups, including non-specific lipid transfer protein (nsLTP) and pathogenesis-related proteins in class 10 (PR-10).

## 2. Case Studies

The following case reports include six patients who were seen in 2022 and 2023 in a tertiary allergy outpatient clinic in Poland. In total, 46 specific Immunoglobulin E (sIgE) assays were conducted using Allergy Explorer 2 (ALEX2) (MacroArray Diagnostics). Out of them, six assays showed positive results for Can s extract and/or the Can s 3 component.

Case 1

A 55-year-old male patient had been referred to an allergist due to recurrent angioedema (AE) attacks. The first AE incident took place at the age of sixteen. After turning 40, AE has been occurring with an average frequency of one incident per year. In 2023, the frequency and intensity of the symptoms increased to one incident per month during the late winter and early spring period (January through April). C1-esterase inhibitor activity and concentration as well as C3 and C4 component protein concentrations in plasma were within normal limits. The patient suffers from hypertension (in May 2023, angiotensin-converting enzyme inhibitor [ACEI] was changed to sartan), left bundle branch block (LBBB), and hyperuricemia. From April to June, symptoms of seasonal allergic rhinitis intensified. Additionally, the patient experienced throat swelling after consumption of moderate amounts of hazelnut. The presence of specific IgE in class 1 for grasses, trees, and mugwort, as well as apples in class 2, was ascertained. A molecular diagnosis showed positive results for pathogenesis-related proteins in class 10 (PR-10), especially: Bet v 1–8.83 kU A/L, Fag s 1–2.26 kU A/L, Cor a 1.0401–1.65 kU A/L, and Cor a 1.0103–3.97 kU A/L (the others are listed in Table 1). Additionally, higher concentrations were also observed in relation to most Timothy grass components (Phl p 1, 2, 5.0101, 6). Furthermore, sensitization was observed in the nsLTP group—Ara h 9 and Cor a 8. Sensitization of the entire cannabis sativa allergen was found—0.31 kU A/L (without an increased level of sIgE to its component, Can s 3).

Case 2

A 10-year-old male patient has been suffering from atopic dermatitis (AD) since the age of five months. The patient was diagnosed with cutaneous mastocytosis, food allergy to cheese, hen’s egg, and cow’s milk protein (positive challenge test), allergic rhinoconjunctivitis, and bronchial asthma. An incident of acute hives occurred after the patient’s mother touched his face with a hand that had previously had contact with cheese. A similar reaction occurred with the dough containing eggs. After the second year of life, the patient started to experience frequent respiratory infections; however, IgA, IgG, and IgM as well as IgG1-4 subclasses were within normal limits. The family history includes allergic rhinitis in both parents and, additionally, asthma in the mother. High levels of sIgE were found in many cross-reactive allergens: profilins, PR-10, nsLTPs, and storage proteins (2S albumins, 7/8S globulins, 11S globulins), including allergens contained in *C. sativa* (Table 1). In this case, in contrast to the previous patient, an increased level of Can s 3 was observed—0.98 kU A/L—while the level of sensitization to cannabis sativa extract was low—0.16 kU A/L.

Case 3

A 29-year-old female patient, presented with redness on the skin of the neckline, elbows, and face with small papules accompanied by itching after contact with onions and leek. She also reported similar skin symptoms after contact with latex gloves and other rubber products. She has been suffering from rhinitis for the last 6 months. Reflux disease symptoms occur quite frequently after meals. The patient has not been using any medications on a permanent basis nor has she suffered from other chronic diseases. In childhood, cow’s milk allergy was suspected based on skin symptoms but no medical documentation from that period is available. The presence of IgEs specific for some nsLTP, namely Art v 3, Mal d 3, Can s 3–3.90 kU A/L (Can s extract—5.59 kU A/L), Zea m 14, Cor a 8, Jug r 3, Api g 2, Api g 6, and Vit v 1, has been ascertained. No sensitization to extracts or components was observed with regard to onion and latex. In complete blood count, peripheral eosinophilia of 430 cells/uL (5.5%) was found. The patient was diagnosed with LTP allergy syndrome and polyvalent inhalant and food allergy.

Case 4

A 20-year-old female patient has been treated for AD since the age of one. Due to the lack of satisfactory results of a standard AD therapy (including cyclosporine), it was decided to start biological treatment with dupilumab. Pollen allergy in the form of rhinitis has been present from early childhood, with the highest intensity between February and May. The patient underwent specific allergen immunotherapy with birch pollen allergens. Seasonal asthma was suspected, but the reversibility test was negative and forced expiratory volume in the first second (FEV1) was 96,7%; forced vital capacity (FVC) was 89,8%. In this case, PR-10, and not nsLTP, is the main group of allergens against which sIgE was detected. The highest levels were observed in relation to Bet v 1- > 50 kU A/L, Cor a 1.0103–47.73 kU A/L, and Aln g 1–45.34 kU A/L. Regarding nsLTPs, sIgE to Ara h 9 and Vit v 1 were slightly increased (0.81 kU A/L and 0.57 kU A/L, respectively). The results regarding another two nsLTP components, Act d 10–10.85 kU A/L and Can s 3–8.51 kU A/L, are noteworthy (Can s as in case 2, low < 0.10 kU A/L). The patient also has a symptomatic food allergy to certain fruits, vegetables, and nuts (laboratory results showed sensitization to Ara h 8, Ara h 9, and Cor a 1.0401), manifested by mouth edema. In laboratory tests, complete blood count (CBC) values were within normal range. here was no history of drug intolerance.

Case 5

A 34-year-old female patient after smoking marijuana at the age of about 16, experienced almost immediate vomiting and drowsiness. Feeling unwell persisted for the next 24 h. The patient considered these symptoms as a result of substance use. In September 2022, the patient tried to smoke marijuana again. Immediately after smoking, confusion, vomiting, diarrhea, dizziness, and shortness of breath occurred, and the symptoms were highly pronounced. They disappeared the next day.

The patient has been diagnosed with polyvalent food allergy. She has had four incidents of anaphylaxis so far. The first occurred in October 2022: 30 min after eating the minced cutlet with sauerkraut and buckwheat, throat swelling, urticaria, and drooping of the mouth appeared. Previously, the patient was exposed to cat allergens. The second incident (February 2023) happened within 30 min after eating a burger with beef, tomato, lettuce, and pickled cucumbers. Possible cofactors were physical activity and high stress. Symptoms during that episode included disturbances of consciousness, urticaria, hoarseness, and throat swelling. The third anaphylaxis (March 2023) similarly occurred 30 min after eating beef kebab with sauerkraut, garlic sauce, and white pita bread. The symptoms were similar to the ones experienced during the previous episode. Possible cofactors included alcohol, exercise, menses, non-steroidal anti-inflammatory drugs (NSAIDs), and oral contraception (on medroxyprogesterone acetate). The last episode of anaphylaxis occurred in October 2023. The patient ate pasta (which she had consumed before without allergic symptoms). The patient complained of throat and neck swelling with aphonia, followed by wheezing and confusion, without skin lesions. Intramuscular adrenaline was injected with partial improvement. Apart from these reactions, the patient reported local symptoms in the oral mucosa, with no apparent systemic symptoms after eating various foods, such as peanuts. She experienced irregular seasonal respiratory symptoms but they were not bothersome and did not require systematic treatment The patient only took antihistamines on an on-demand basis. Comorbidities included Attention Deficit Hyperactivity Disorder (ADHD) with no other conditions reported. Molecular diagnostics revealed high sIgE levels against most allergens from the PR-10 and nsLTP families. Regarding PR-10, the highest sIgE levels were seen for Bet v 1–48.37 kU A/L, Cor a 1.0103–36.42 kU A/L, and Fag s 1–22.32 kU A/L (also increased for Mal d1, Dau c1, Aln g 1, and Cor a 1.0401). In the case of nsLTPs, sensitization to most allergens was found, especially to celery (Api g 2–41.30 kU A/L, Api g 6–2.89 kU A/L), Can s 3–35.99 kU A/L (Can s 5.15 kU A/L), Pru p 3–24.05 kU A/L, Mal d 3–22.80 kU A/L, and Pla a 3–19.33 kU A/L. The remaining results regarding nsLTPs are shown in Table 1.

Case 6

A 35-year-old male patient presented with nasal itching and congestion, breathing difficulties, and excessive lacrimation. Urticaria lesions were observed in association with paracetamol and ibuprofen ingestion. In childhood, he was treated for allergic rhinitis and asthma. The symptoms of asthma recurred in 2021 in the form of tightness in the chest and breathing difficulties. Allergic rhinitis occurs in summer. After consuming onions, he developed oral allergy syndrome (OAS) symptoms. Moreover, he observed shortness of breath after eating tuna with onion. He declined to have any pets. Allergy family history was negative. He has been working as a salesman and reports no occupational hazards in the past. He has been treated with long-acting beta-agonists (LABA), inhaled and topical glucocorticosteroids, and antihistamines. High levels of sIgE to multiple D. pteronyssinus and D. farinae components were observed (Der f 1, Der f 2, Der p 2, Der p 5, Der p 7, Der p 23). sIgE for nsLTPs were also increased for Can s 3–8.43 kU A/L (although 0.77 kU A/L for Can s), 3.76 kU A/L for Cor a 8, 2.56 kU A/L for Api g 2, and 1.46 for Zea m 1. No sensitization to onion and tuna was found.

Cases descriptions are summarized in Table 2 with regard to symptoms, presence of atopy and sensitization to PR-10, nsLTP, cannabis extract and Can s 3 component.

## 3. Pathogenesis

Four allergens of cannabis sativa have been identified so far with the non-specific lipid transport protein (nsLTP) Can s 3 being considered as the essential cross-reacting allergen [6]. The major biological role of nsLTPs seems to be the transfer and deposition of lipids for the assembly of complex barrier polymers on the surface of plant tissues and organs [7]. The structure of nsLTPs results in resistance to proteolytic digestion and thermal processing [8].

Thus, immunogenicity will be maintained even after thermal processing of the food. LTPs are essentially localized in the pericarp of the fruits [9]. Sensitization to nsLTPs can occur in several different ways: through cutaneous exposure, via the gastrointestinal tract, or inhalation. The inhalation way of sensitization may apply not only to cannabis but also to some fruit allergens (e.g., peach) [7,10]. Cases have been described where people who had occupational contact with peaches had symptoms indicating an allergy to Pru p 3 [11,12]. Specific bronchial provocation tests with extracts of peach leaf and a nasal challenge with peach peel extract induced respiratory symptoms [11,12]. Sensitization and allergy to LTPs predominate in areas of southern Europe but can be observed in other geographical locations as well. Already, in 2013, a Belgian study showed that illicit cannabis abuse can result in CA with cross-reactivity or cosensitization due to nsLTPs [13]. The prevalence of LTP sensitization is not comparable and is lower in patients recruited outside the Mediterranean area, defining them as minor allergens, which requires further research due to the increasing incidence of sensitization [8]. nsLTP sensitization represents the variability of its clinical presentation, which may range from mild contact urticaria to anaphylaxis [14]. The controversial issue is what is responsible for primary sensitization regarding LTP allergies. Peach (Pru p 3) is the most often mentioned allergen, although Can s 3 may also be related [10].

Cannabis may also be a cofactor of the generalized hypersensitivity reaction [15]. This raises a reverse question—what may be the cofactors causing allergic reactions to cannabis, as shown in case 5? Various factors can be considered in this context including physical exercise, alcohol, and drugs (NSAIDs, oral contraceptives). The multitude of allergic reactions indicates the occurrence of not only cofactors but also cross-reactions. However, the existence of pork–cat syndrome can be ruled out due to the lack of sIgE on cat serum albumin (Fel d2) and pork albumin (Bos d 6) [16].

Case 2 addresses the issue of LTP sensitization in the pediatric population. As shown in the Italian study by Indolfi et al., in the years 2010–2020 there was a statistically significant increase in the number of LTP-sensitized patients. According to the cited authors, the most commonly sensitizing LTP was Pru p 3 which affected 46% of the population. In second and third place were Jug r 3 (32.4%) and Art v 3 (31.9%), respectively [17]. The patient we describe presents sensitization to most allergens from the nsLTP family. The results of the component-resolved diagnostics (CRD) also revealed high levels of IgE sensitization to profilins, PR-10, and storage proteins. However, the symptoms of allergies indicate products from other families. Indeed, in this case, high levels of sIgE in animal milk and hen’s eggs correlate with intense allergy symptoms.

In recent years, further immunogenic proteins have been discovered. A cannabis homolog of Bet v 1 (PR-10 homolog) and recombinant cannabis–profilin have been indexed by the WHO/IUIS Allergen Nomenclature Subcommittee as Can s 5 and Can s 2, respectively [18,19]. In total, 8 of 113 CA patients in a Northwestern European region demonstrated a positive sIgE result (≥0.10 kU A/L) for oxygen-evolving enhancer protein 2 (OEEP2), involved in plant photosynthesis known as Can s 4 [18,20]. A study by Loblundo et al. showed that in extracts from distinct varieties of cannabis, there is the presence of at least 50 different proteins that share homology with known allergens (including airway, food, contact allergens, and those with the capability to sensitize through multiple routes) [21].

However, this does not mean that all CA patients are sensitized to Can s 3. In a study by Decuyper et al., 42 of 120 patients did not show this sensitization [15]. In this context, our case no. 1 supports the theory that other cannabis allergens may lead to sensitization.

In a study by Mamone et al., hemp protein isolate from a defatted hemp meal was subjected to proteomic analysis. Interestingly, any of the known hemp allergens either before or after in vitro gastroduodenal–BBM digestion were detected. However, six of the identified peptides arose from Z-serpins (protease inhibitors), as identified by homology with the wheat and barley counterparts, which are possible triggers of IgE-mediated food allergies [22]. In what regards potential cross-reactivity, marijuana users are more likely than non-users to be sensitized to molds (Alternaria alternata), dust mites (D. farinae and D. pteronyssinus), plants (ragweed, ryegrass, Bermuda grass, oak, birch, and peanut), and cat dander [23].

## 4. Symptoms

The symptoms of CA present a broad spectrum. The main distinction is the route of intake of the allergen, which includes smoking, ingestion, the inhalation of pollen, and direct contact. It should also not be forgotten that contact with the allergen may occur as a result of the passive inhalation of cannabis smoke, which will be described in more detail below. Due to the limited number of studies, it is difficult to provide more precise epidemiological data regarding not only the number of patients affected but also the frequency of symptoms. The most common symptoms among suspected CA (*n* = 445) were urticaria or angioedema (51.6%), nasal congestion (43.2%), rhinitis (45.3%), and cough (41.7%) [5]. These are relatively mild symptoms, but it is important to remember that the course of anaphylaxis may be violent. Gilbert et al. [24] report a case of anaphylaxis after intravenous administration of cannabis while Cabrera-Freitag et al. [25] described anaphylaxis induced by passive second-hand exposure to *C. sativa* cigarette smoke. In the first case, there was generalized urticaria, difficulty breathing, and wheezing. In the second, the patient reported an episode of generalized urticaria, facial swelling, difficulty breathing, wheezing, and dizziness. Both patients had a positive skin prick test (SPT) result to protein extract from *C. sativa* buds and Pru p 3. Stepaniuk and Kanani presented a case of selective cannabis strain allergy [26]. Another patient suffering from second-hand CA is a 6-year-old boy associated with chronic worsening of asthma [27]. After removing cannabis from his environment, the patient reported significant improvement in all symptoms. De Silva et al. in 2015 described food-dependent exercise-induced anaphylaxis following the inhalation of cannabis along with the ingestion of wheat and coconut derivatives. Interestingly, the patient smoked cannabis without any symptoms subsequently provided wheat was not consumed in relation to exertion. It was the first report in the literature of this drug implicated as a co-factor [28]. Another interesting case report is about contact urticaria with cannabis leaves and the second time, the same patient complained of rhinoconjunctivitis when exposed to marijuana smoke [29]. Anaphylaxis can occur after eating yogurt with hemp seeds [30]. This patient also marked reactivity to stone fruits (cherry, nectarine, peach) and hazelnut in SPT which suggests nsLTP cross-reactivity. The result on the cannabis sativa component is interesting in case 4. There was probably a cross-reaction between kiwi fruit, peanut, or grape. Contact urticaria to cannabis sativa occurred in a patient working in *C. sativa* harvesting [31]. Another case also presented cannabis sensitization and allergy resulting from the above-mentioned passive exposure to cannabis smoke and/or indirect cutaneous transmission [32]. The last symptom of CA worth mentioning is rhinoconjunctivitis caused by cannabis sativa pollen [33]. As can be observed, CA symptoms present a wide spectrum of symptoms affecting all systems, including skin and mucosal, gastrointestinal, upper and lower respiratory, neurological, and cardiovascular.

Most of the described symptoms of CA are based on case reports in the literature, and thus Table 3 summarizes the occurrence of symptoms along with their severity and treatment in patients diagnosed with CA [15,25,26,29,30,32,33]. Since the case reports were published over a few decades and different grading systems were used for the assessment of symptoms, we have set to employ two different current grading scales to unify and compare the clinical picture of anaphylaxis described in different reports. We employed a severity grading system of food-induced acute allergic reactions developed by Błażowski et al. in 2021 [34], which includes symptoms of anaphylaxis that were omitted in previously used classifications. Moreover, Table 4 compares the incidence of anaphylaxis after cannabis on two anaphylaxis-grading scales.

### Occupational Hazards

It is worth mentioning the occupational hazards associated with cannabis cultivation. Both legal and illegal cannabis cultivation release cannabis pollen that may have immunogenic properties. A Swiss study over a 3-decade period (1990–2020) showed that the cannabis pollen season starts earlier, lasts longer, and is more intense [36]. In southeastern Spain, the cannabis pollen season occurs between June and August. Back trajectory analysis showed the pollen origin to be mainly from the local region and long-distance transport from Africa was infrequent which emphasizes the theory of expanding cannabis crops [37].

In a study by Sack et al. 21, 31 (71%) employees in an indoor cannabis grow facility reported one or more work-related symptoms: respiratory (the most frequent), ocular, nasal, or dermal. However, this study has a limitation due to the high incidence of recreational cannabis use among these workers [38]. Another issue is a small group of participants on whom health measurements were made. Five of the ten workers had borderline or abnormal fractional exhaled nitric oxide (FeNO), and seven (70%) had abnormal spirometry. Five (50%) participants demonstrated cannabis sensitization to one or more strains on SPTs. In another study, including occupational cannabis exposure in police force personnel, 34 of the 81 participants (42%) reported respiratory and/or cutaneous symptoms [39]. On the other hand, all SPTs with the nsLTP-rich cannabis extract yielded negative results, as well as basophil activation tests (BATs) for crude cannabis extract and recombinant Can s 3 (rCan s 3)—except in one symptomatic case that demonstrated an isolated and borderline result for rCan s 3. There was also no significant difference between the groups with and without symptoms of cannabis exposure in terms of allergenic sensitivities to house dust mites, components of different endemic pollen, and three different molds. In contrast to the earlier study, only 3 of 81 participants reported asymptomatic recreational use of cannabis dating back more than 12 months. Another study presents case reports of occupational exposure in police personnel and forensic services to cannabis resulting in a persistent, urticated rash [40]. None of the patients reported recreational use of cannabis.

The above-mentioned studies lead to the consideration of several topics. Firstly, whether the symptoms of occupational exposure were immunological or non-immunological was considered. Secondly, could previous exposure to cannabis cause the acquisition of immune tolerance to this allergen? Not only cannabis pollen can have a negative impact on health. For example, workers in cannabis cultivation can be exposed to high bioaerosol concentrations which can cause pulmonary infections and respiratory diseases such as occupational asthma [41].

## 5. Diagnostics

Currently, there are no guidelines for the diagnosis of cannabis allergy. SPT-containing standardized cannabis allergen has not been created yet. Legal regulations in individual countries make it difficult to use marijuana samples in diagnostics. In Poland, the permissible THC content in cannabis products is 0.3% while the THC content in marijuana has shown an upward trend over the years, reaching as much as 13.88% in 2019 in the United States [42,43]. Component diagnostics, used in the context of allergens from various sources, represent an invaluable role [44]. In vitro tests for cannabis-specific IgE can be performed by using ALEX2 (commercially available in Europe) or ImmunoCAP (available upon request for research purposes) [45].

Decuyper et al. explored the performance of five cannabis diagnostic tests among patients with cannabis allergy: specific IgE (sIgE) hemp, sIgE and basophil activation test (BAT) with a recombinant Can s 3 protein from cannabis sativa (rCan s 3), BAT with a crude cannabis extract, and a skin prick test (SPT) with a nCan s 3-rich cannabis extract [15]. The highest sensitivity (82%) was for sIgE hemp (but the lowest specificity in the whole group—32%), whereas specificity (87%) was for sIgE rCan s 3. In another study, prick tests and IgE for cannabis had good sensitivity (92 and 88.1%, respectively) and specificity (87.1 and 96%) for cannabis sensitization [46]. Unlike the previous subjects in Decuyper et al.’s research consisting of CA patients, in this one, asthmatic patients sensitized to pollen, and those sensitized to tobacco, tomato, and latex, were selected. Thus, the sensitivity and specificity of these tests cannot be compared.

Another test that can be used for CA diagnosis is a bronchial challenge, first described in 1991 [47]. In 2011, Armentia et al. conducted another bronchial challenge with cannabis extracts which were diluted to 0.005 mg/mL, 0.05 mg/mL, 0.5 mg/mL, 1 mg/mL, and 5 mg/mL [46].

Specific IgE to *C. sativa* in patients with CA was significantly higher as compared to food-allergic non-cannabis allergic patients [13]. Among cannabis nonsmokers, the prevalence of sensitization to *C. sativa* in SPT was 5% (19/379) whereas for smokers, the prevalence was 14.6% (21/144) [OR 3.2 (1.6–6.2), *p* < 0.001] and even higher in frequent or regular smokers (4/22, 18.2%) [48]. A study conducted in India indicated that cannabis sativa was one of the most common allergens resulting in positive sensitization in SPTs [49]. In total, 17 of 50 subjects were presented with a wheal of 3.52 mm diameter. To sum up, it should be taken into account that patients who demonstrated SPT reactivity to *C. sativa* extracts did not necessarily exhibit IgE reactivity to *C. sativa* extracts and some SPT-negative patients may demonstrate IgE reactivity to *C. sativa* extracts [50]. Referring to the above-quoted survey among allergists, frequency of performing skin prick testing for suspected cannabis allergy varied significantly and was reported by 25.3% to 71.4% of respondents. In vitro testing was infrequent (6.8% of the respondents) [5].

## 6. Cannabis and Atopic Diseases

**Figure 1:**
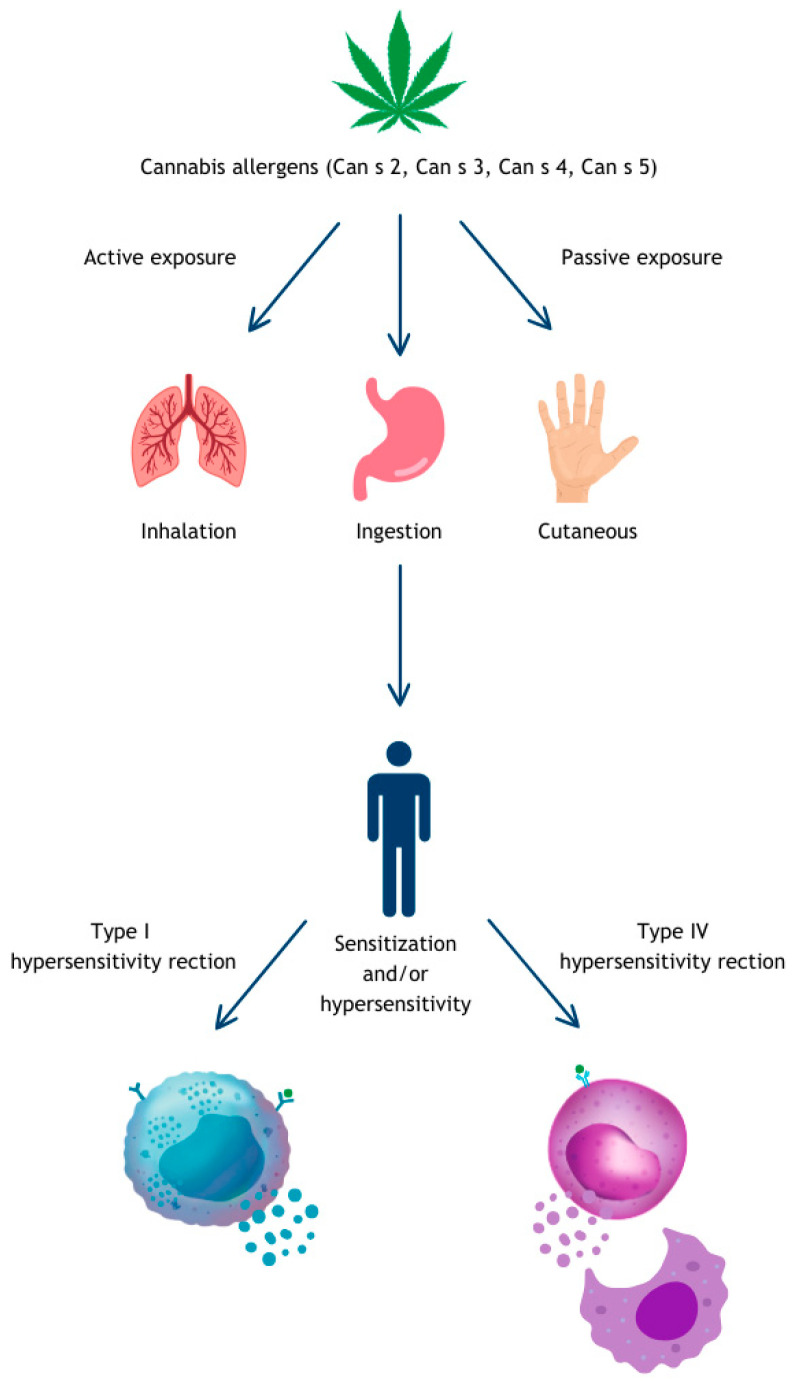
Mechanism of sensitization to cannabis.

Most cannabis hypersensitivity reactions are mediated by an allergic mechanism, either type 1 or type 4 [51]. Symptoms of cannabis allergy or hypersensitivity may result from different routes of exposure – Figure 1. These facts justify investigation of a possible relationship between cannabis sensitization and diseases such as asthma, allergic rhinitis, or atopic dermatitis.

### 6.1. Asthma

In a large study consisting of 227,451 US children between 2011 and 2019, among youth ages 0–5, relatively, pediatric asthma prevalence decreased significantly (*p* < 0.05) in states with recreational use (RCL), with medical use (MCL), and in states without cannabis legalization. On the other hand, among youth ages 12–17, the prevalence of asthma increased in states with cannabis legalization, and particularly in states with RCL, the increase was statistically significantly greater compared with states without legalization (*p* = 0.028) [52]. Another study conducted on American public high school students showed that the odds of lifetime asthma were significantly higher for groups using both exclusive cannabis (AOR = 1.17) and dual E-cigarette/cannabis use (AOR = 1.17) and also when cigarettes were added to the above-mentioned two substances (AOR = 1.14) [53]. Among 178 young adults with asthma, caregiver nicotine use was correlated with caregiver cannabis use (r = 0.30, *p* < 0.001) and friend nicotine use was correlated with friend cannabis use (r = 0.43, *p* < 0.001) [54]. Studies indicate not only an increase in the risk of asthma but also worse test results in asthmatics. Cannabis users with uncontrolled asthma exhibited poorer mini asthma quality of life questionnaire (mAQLQ) (*p* < 0.001) and Nijmegen scores (*p* < 0.001) and a higher frequency of hyperventilation (*p* < 0.01) compared to those who never used cannabis [55]. A Norwegian study showed that the odds ratio for a current cannabis young-adult user to fill prescriptions for asthma medication was 1.71 (95% CI: 1.06–2.77; *p* = 0.028) [56]. The above-mentioned studies concern a specific age population, more specifically young people. However, there is also a study involving the adult population. Regular cannabis use in this group was significantly associated with greater risk for asthma both in the with and without tobacco co-use subgroups [57].

All of the articles mentioned discuss clinically symptomatic allergies. There is a lack of evidence in the literature regarding cannabis sensitization and cannabis exposure-related asthma. Therefore, we suggest that the relationship between atopic diseases and cannabis sensitization/allergy be further investigated, and that research be expanded in the context of allergen families such as nsLTP or PR-10. According to Esteban-Gorgojo et al., among pediatric patients with asthma, LTP sensitization is a risk factor for having a concomitant food allergy diagnosis (*p* = 0.016, OR: 3.064, RR: 2.512), whereas this association was not found for profilin sensitization [58]. This is especially significant in the context of the most extensively described component Can s 3, which belongs to nsLTP.

### 6.2. Atopic Dermatitis

In our report, we reviewed 46 results performed with the ALEX2 test over a nearly two-year period. Two of the six patients (33%) showing sensitization were diagnosed with AD. We understand that this is a very limited group, and therefore we refer to the available literature. In a study by Čelakovská et al., among 100 AD patients examined with the use of ALEX2, 12 showed sensitization to Can s [59]. We may observe some interesting results regarding the potential positive impact of cannabis on inflammation and symptoms. In Canada, atopic dermatitis is one of the most common dermatologic conditions being treated with topical cannabis [60]. Dietary hempseed oil and CBD may have a beneficial effect on the symptoms and immune pathways of AD, respectively [61].

## 7. Pro- or Anti-Inflammatory?

The human endocannabinoid system is involved in the control of many relevant physiological processes. Cannabinoid receptors, CB1 and CB2, are one of the main components of this system [62]. The concentration of CB1 receptors is higher in the central nervous system, whereas CB2 receptors are primarily found in immune cells but this does not mean that CB1 does not affect them as well [62]. THC is a partial agonist of CB1 and CB2 receptors, primarily targeting the cannabinoid CB1 receptor [63]. In contrast to THC, cannabidiol (CBD), a nonpsychotropic component, binds very weakly to CB1 and CB2 receptors [64]. It is believed that THC has an immunosuppressive effect, while CBD has an anti-inflammatory effect.

An issue that requires further research is the exact mechanism of cannabis’s effect on cytokines. Cannabis extract of the high CBD *C. sativa* strain reduces the level of IL-8 and IL-6 in the lung epithelial cell model [65]. Moreover, CBD alone or combined with THC in most studies has a positive effect in reducing inflammation (as reflected by the secretion of TNF-α, IL-1β, IL-6, IFN-γ) in various disease states. This effect was not observed whereas THC alone was used as an intervention [66]. On the other hand, in the meta-analysis, most studies did not show a significant effect of cannabinoids on IL-6 among cannabis users [67]. Cannabis smoke exposure modifies the percentage of innate immune cell populations and alters the percentage of lymphoid cells in the lungs of mice. Interestingly, the changes in bronchoalveolar lavage (BAL) cytokines were more pronounced when mice were exposed to CBD compared to THC variety [68]. In a study by Vuolo et al., 10 mg/kg of CBD i.p. significantly decreased the number of eosinophils in the lung tissue in a murine model of ovalbumin-induced allergic asthma when compared to mice sensitized and challenged with ovalbumin [69]. Moreover, 5 and 10 mg/kg CBD decreased measured cytokines (IL-4, IL-5, IL-13, eotaxin) compared to the OVA model. Lower cytokine levels were observed at the lower dose of CBD. Also in mice, WIN55212-2, a non-selective synthetic cannabinoid, reduces peanut-allergic sensitization by lowering levels of peanut-specific IgE and IgG1 and promotes the generation of peanut-specific CD4+ CD25high FOXP3+ Treg cells [70]. Another cannabinoid, beta-caryophyllene (β-CP), when administered topically, causes pruritus in mice and also contributes to an increase in total IgE in serum. In contrast, CBD did not induce scratching, did not yield any change in serum IgE, and was comparable to mice treated with vehicle (acetone) [71].

## 8. Therapeutic Opportunities

The issue of treatment is challenging to discuss due to the very small amount of the known literature on this topic. Currently, the best treatment choice is avoidance. There is, however, a report of successful attempts to control the frequency and severity of cannabis-induced anaphylaxis with omalizumab [72]. At this point, it is worth noting that omalizumab in early 2024 gained the US Food and Drug Administration (FDA) approval for the treatment of food allergies in patients aged 1 year and older [73]. Alternative treatment strategies included using—with satisfactory results—antihistamines and corticosteroids in the above-described patient with selective cannabis strain allergy [26]. The use of subcutaneous immunotherapy in patients suffering from bronchial asthma and allergic rhinitis that exacerbates during the pollination period of cannabis also looks promising [74]. The development of forms of allergen immunotherapy and their effectiveness in desensitization encourages the search for new possibilities in treatment [75]. Developing branches of immunotherapy, though reported in the form of oral mucosal immunotherapy (OMIT) by using toothpaste for the treatment of peanut allergy, demonstrate that there is also a need to focus not only on the search for effective treatment of cannabis allergy symptoms but also their prevention [76]. Another approach is to use genetic engineering. There have been reports of cutting allergen genes with clustered regularly interspaced short palindromic repeats (CRISPR) technology. CRISPR technology has been applied to edit allergen genes in cats, hen’s eggs, soybean, wheat, peanut, and cow’s and goat’s milk [77]. Undoubtedly, biological treatment as well as genetic engineering are highly advanced technologies. However, there is a lack of clinical research on basic allergy treatment, such as the use of antihistamines or inhaled drugs for respiratory symptoms.

## 9. Conclusions

**Figure 2:**
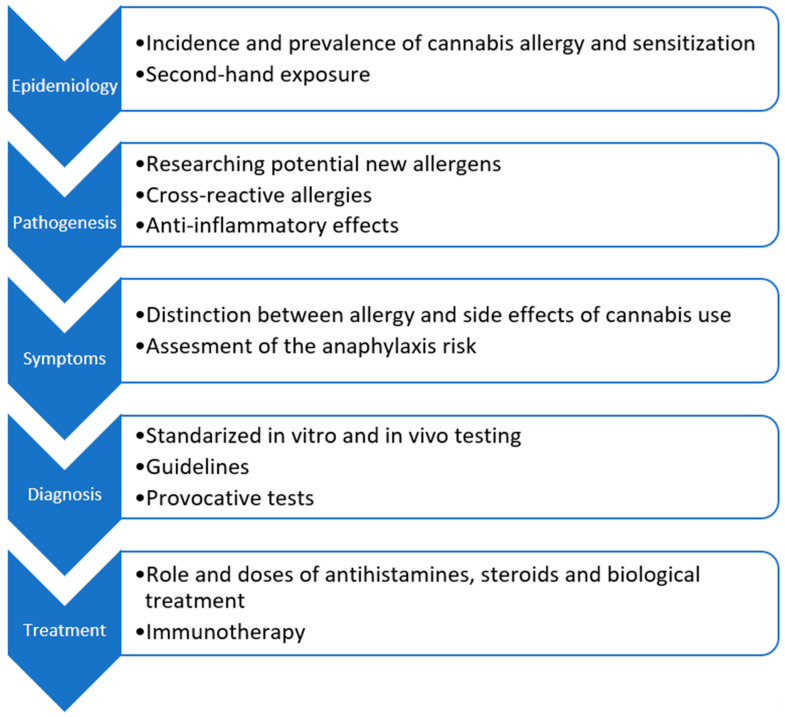
Future directions on research of cannabis allergy and sensitization.

We presented six different patients with sensitization to cannabis and/or the main sensitizing protein, Can s 3. They became the basis for creating the current literature review, where most of the studies had been published less than 5 years ago. As presented in Figure 2, we identify and suggest possible future directions of research on cannabis allergy and sensitization. Starting with the epidemiological issues, we emphasize that the problem of sensitization to cannabis allergens in the population may be broader than it first appears, so it is essential to propagate in commercial testing the availability of not only the entire cannabis allergen extract but also the individual components. Recent studies have shown that there are new components in addition to Can s 3 that can cross-react with other allergens, as in the case of Can s 5 and Can s 2. Thus, we moved on to aspects of the pathogenesis of cannabis allergy, where we pointed out that it is a complex allergy, also based on cross-reactions. We also raised the discussion of cannabinoid receptors in the context of THC and CBD, which may hint at their dualistic nature of function. Of note, although sensitization to cannabis occurs in AD patients, topical use of cannabinoids may have anti-inflammatory effects. Symptoms involving multiple systems that have been widely described in the literature as well as in this review were summarized in the form of tables. Their widespread influence is noteworthy, ranging from local reactions to an anaphylactic shock ending tragically. In the context of diagnosis, although sIgE for cannabis allergens can be detected, there is a lack of broader validation of further diagnostic methods commonly used in clinical practice such as SPT or challenge tests. Interestingly, they have already been described in the literature for a relatively long time, such as the bronchial challenge in 1991. We believe that a holistic view of the topic and creating bullet points of future directions can encourage researchers and clinicians to lean into this issue.

## Figures and Tables

**Table 1:** Serum levels of IgE specific to cannabis sativa extract and nsLTP allergenic components from different sources in presented patients.

	Pla a 3	Art v 3	Can s	Can s 3	Ara h 9	Zea m 14	Act d 10	Mal d 3	Pru p 3	Vit v 1	Api g 2	Api g 6	Sola l 6	Cor a 8	Jug r 3	IgE, Total
#1			0.31		0.12									0.14		67
#2	0.58	8.36	0.16	0.98	1.36	1.77	1.59	4.22	1.7	3.04	0.78	0.32	0.77	1.16	8.48	813
#3		0.89	5.59	3.90	0.24	3.18		0.57	0.17	1.08	2.34	6.48		2.68	0.44	120
#4				8.51	0.81		10.85			0.57						490
#5	19.33	5.92	5.15	35.99	1.42	6.65		22.80	24.05	5.98	41.30	2.89	6.89		2.85	807
#6	0.5	0.72	0.77	8.43	0.20	1.46		0.88	0.26		2.56		0.36	3.76		73

Note: positive sIgE result ≥ 0.10 kU A/L.

**Table 2:** Case studies summary.

	Age	Cannabis Allergy Symptoms	Atopy	IgE to PR-10 Allergens	IgE to nsLTP Allergens	IgE to Can s	IgE to Can s 3
#1	55	-	+	+	+	+	-
#2	10	-	+	+	+	+	+
#3	29	-	+	-	+	+	+
#4	20	-	+	+	+	-	+
#5	34	+	+	+	+	+	+
#6	35	-	+	-	+	+	+

**Table 3:** Frequency of generalized symptoms in cannabis allergy, based on published cases reviewed in manuscript (according to grading scale by Błazowski et al. [34]).

System	Symptoms	*n* (%) Total = 33 (100)
Skin and mucosal	Pruritus	9 (27)
	Generalized urticaria	22 (67)
	Angioedema (not laryngeal)	14 (42)
Gastrointestinal	Oral/palatal pruritus, oral/palatal tingling	1 (3)
	Nausea/drooling	3 (9)
	Crampy abdominal pain	1 (3)
	Sudden and/or recurrent vomiting	3 (9)
Upper respiratory	Nasal symptoms	19 (58)
	Feeling of difficulty in breathing	3 (9)
Lower respiratory	Sudden, repetitive cough	2 (7)
	Chest tightness/dyspnea	23 (70)
	Mild to moderate bronchospasm	4 (12)
Neurological	Presyncope (dizziness/weakness)	1 (3)
	Confusion/somnolence	1 (3)
Cardiovascular	Sudden, relevant hypotension	1 (3)

**Table 4:** A comparison of the severity of allergic reactions in published cannabis allergy cases reviewed in the manuscript, as assessed using 2 different grading systems proposed by Błażowski et al. [34] and the World Allergy Organization anaphylaxis-grading scale [35]. A total number of 33 cases have been found in the literature. The numbers and percentages of cases that have been attributed given grades according to each grading system are presented.

Błażowski et al. [34] (*n* (%))	WAO (*n* (%))
I—3 (9)	I—1 (3)
II—28 (85)	II—10 (30)
III—2 (6)	III—16 (49)
IV—0	IV—5 (15)
	V—1 (3)

## Data Availability

The retrospective data contained in this manuscript are available from the corresponding author (M.K.) upon reasonable request, with respect to personal data protection laws.

## Abbreviations

Abbreviations for allergen components used in the manuscript.

**Abbreviation**	**Allergen Source (Latin Name)**	**Common Name**	**Group**
Act d 10	*Actinidia deliciosa*	Green kiwi fruit	nsLTP
Aln g 1	*Alnus glutinosa*	Alder	PR-10
Api g 2	*Apium graveolens*	Celery	nsLTP
Api g 6	*Apium graveolens*	Celery	nsLTP
Ara h 8	*Arachis hypogaea*	Peanut	PR-10
Ara h 9	*Arachis hypogaea*	Peanut	nsLTP
Art v 3	*Artemisia vulgaris*	Mugwort	nsLTP
Bet v 1	*Betula verrucosa*	European white birch	PR-10
Can s	*Cannabis sativa*	Hemp	
Can s 3	*Cannabis sativa*	Hemp	nsLTP
Cor a 1.0103	*Corylus avellana*	Hazelnut	PR-10
Cor a 1.0401	*Corylus avellana*	Hazelnut	PR-10
Cor a 8	*Corylus avellana*	Hazelnut	nsLTP
Dau c 1	*Daucus carota*	Carrot	PR-10
Der f 1	*Dermatophagoides farinae*	American house dust mite	Cysteine protease
Der f 2	*Dermatophagoides farinae*	American house dust mite	NPC2 family
Der p 2	*Dermatophagoides pteronyssinus*	European house dust mite	NPC2 family
Der p 23	*Dermatophagoides pteronyssinus*	European house dust mite	Peritrophin-like protein domain
Der p 5	*Dermatophagoides pteronyssinus*	European house dust mite	Group 5/21 allergen
Der p 7	*Dermatophagoides pteronyssinus*	European house dust mite	Bactericidal permeability-increasing-like protein
Fag s 1	*Fagus sylvatica*	European beech	PR-10
Fra a 1	*Fragaria ananasa*	Strawberry	PR-10
Fra a 3	*Fragaria ananasa*	Strawberry	nsLTP
Jug r 3	*Juglans regia*	English walnut	nsLTP
Mal d 1	*Malus domestica*	Apple	PR-10
Mal d 3	*Malus domestica*	Apple	nsLTP
Par j 2	*Parietaria judaica*	Pellitory of the wall	nsLTP
Phl p 1	*Phleum pratense*	Timothy	Beta-expansin
Phl p 2	*Phleum pratense*	Timothy	Grass group II/III
Phl p 5.0101	*Phleum pratense*	Timothy	
Phl p 6	*Phleum pratense*	Timothy	
Pla a 3	*Platanus acerifolia*	London plane tree	nsLTP
Pru p 3	*Prunus persica*	Peach	nsLTP
Sola l 6	*Solanum lycopersicum*	Tomato	nsLTP
Tri a 14	*Triticum aestivum*	Wheat	nsLTP
Vit v 1	*Vitis vinifera*	Grape	nsLTP
Zea m 14	*Zea mays*	Maize	nsLTP
